# Growth Hormone and Reproduction: Lessons Learned From Animal Models and Clinical Trials

**DOI:** 10.3389/fendo.2019.00404

**Published:** 2019-06-26

**Authors:** Carlos Dosouto, Joaquim Calaf, Ana Polo, Thor Haahr, Peter Humaidan

**Affiliations:** ^1^Obstetrics, Gynecology and Reproductive Medicine, Hospital de la Santa Creu I Sant Pau- Fundació Puigvert, Barcelona, Spain; ^2^Faculty of Medicine, Autonomous University of Barcelona, Barcelona, Spain; ^3^The Fertility Clinic Skive Regional Hospital, Skive, Denmark; ^4^Faculty of Health, Aarhus University, Aarhus, Denmark

**Keywords:** growth hormone, infertility, poor ovarian response, POSEIDON, IVF

## Abstract

Growth Hormone (GH) has been considered as a therapeutic option to increase the number of growing follicles during Assisted Reproductive Technology (ART) for more than 30 years. In this review the biological rationale for therapeutic GH usage is explained through evidence in animal models, aiming to put this into a clinical context. First, we explain the GH—Insulin like Growth Factor (IGF)-1—gonadal axis and its role in reproduction. Evidence suggests that GH can stimulate the secretion of IGF1 not only in the liver but also in the peripheral target structures, including the ovary. Moreover, IGF-1 can be secreted locally under the influence of stimuli other than GH. In the case of the ovary, steroid hormones, gonadotropins or the combination of both seems to be involved. Even more interesting, the ovary itself can secret GH locally and exert a paracrine action modulating the intracellular signaling pathway of GH, i.e., not by the systemic pathway where GH binds to the extracellular domain of the GH receptor. Finally, these aspects from animal models are put into clinical perspective by discussing results and shortcomings of studies and meta-analyses in order to put forth the state-of-the-art rationale for therapeutic GH usage in modern ART.

## Introduction

GH is a monomeric protein secreted by the pituitary with a high molecular similarity to other lactogenic hormones like prolactin and placental lactogen. In the anterior pituitary gland, the secretion by the somatotroph cell is regulated by both stimulatory peptides [e.g., Growth Hormone Releasing Hormone (GHRH)] and inhibitory (e.g., Somatostatin) peptides. The secretion takes place in a pulsatile way that combines short-term variability of spikes of irregular amplitudes with a clear circadian increase, coinciding with the late non-Rapid Eye Movement (REM) periods, probably mediated by dopamine related neurotransmitters (1, 2). This complicates the determination of optimal plasma levels. The action of GH is exerted through its binding to the extracellular domain of a complex membrane receptor. In contrast to dimeric glycoproteins like gonadotropins, two receptors are needed in order to establish a trimeric structure composed by two membrane receptors and the GH molecule. Thus, three recognition processes are needed for an effective downstream activation: receptor-to-receptor and agonistic GH molecule to each of the receptors to form the activated GH trimeric complex. This complex relationship between the hormone and the target organ makes the process of activation vulnerable to different mutations, causing different downstream effects such as the clinical diversity in the different phenotypes of e.g., dwarfism (3, 4). The classic paradigm establishes that pituitary GH acts on its hepatic receptors and stimulates the secretion of somatomedins or Insulin-like growth factors (IGFs): insulin like growth factor 1 and 2 (IGF-1 and IGF-2). These are molecules sharing near 50% homology with pro-insulin. However, IGF-1 and IGF-2 seem to have different roles. While IGF-1 is considered the mediator of the classical biological actions on growth, development and cellular proliferation, IGF-2 is relevant in the regulation of perinatal development. The secretion of IGFs is induced by GH mediated activation of single copy genes. IGF mRNAs have been detected in several target tissues, and at the same time both IGF types exert a negative feedback at the hypothalamic level maintaining basal steady levels of GH (1). Both circulating and local bioavailability is regulated by high affinity binding proteins which fine-tune their local action (5).

## New Evidence from Animal Models

In the last decade the old paradigm described above has been challenged by new evidence obtained in genetically manipulated research animals, introducing new elements of complexity to be taken into account when interpreting the role of GH in any peripheral structure, especially in the ovary. As reviewed thoroughly by others (6), GH can stimulate the secretion of IGF-1 not only in the liver but also in peripheral target structures (6). Moreover, IGF-1 can be secreted locally under the influence of stimuli other than GH. In the case of the ovary, steroid hormones, gonadotropins or the combination of both can be involved. Finally, the ovary itself can secret GH locally and exert a paracrine action, modulating the intracellular signaling pathway of GH and this occurs without binding to the extracellular domain of the membrane GH receptor. This is especially relevant since, contrary to the pituitary secretion, the ovarian secretion of GH takes places in a regular, non-pulsatile, non-circadian pattern.

In GHR knockout mice, circulating GH levels are high and IGF-1 levels are low (7). All studies on blocking or impairing the action of GH on its receptor report a delay in puberty, a significant reduction in litter size (a mean from 6, 7 in wild type animals to 2, 7 in transgenic) (8), and a corresponding delay in the exhaustion of the follicular pool (3). However, the genetically modified animals are fertile and deliver small litters of healthy animals. The experimental data show that this decrease in litter size is the consequence of a reduction in ovulation rate rather than problems related to implantation failure or early embryo loss. Histological examination of the ovaries shows an increase in primordial or primary follicles and a decrease in the number of healthy and growing antral or pre-ovulatory follicles (7–10). It is difficult to establish to which extent this is the result of abnormal GH signaling or its immediate downstream mediator, i.e., a decrease in local IGF-1 secretion. Interestingly, the negative effects seen in the abovementioned studies can be reverted by the administration of IGF-1 (11).

A suitable model to clarify the specific role of IGF-1 is the IGF-gene knockout mouse (12). In these mice, the lack of expression of IGFs results in dwarfism and infertility. The female mutated animals fail to ovulate either spontaneously or under the influence of gonadotropins, proving the importance of IGF1 in the progression of cohorts from primordial and primary stage to recruitable secondary follicles and in the sensitivity to gonadotropins during the process of selection and follicular growth. Interestingly, the histological observation shows an increase in primordial and primary follicles as compared to the wild type animal and an absence of antral follicles. These findings reinforce the idea of a crucial role for IGF in the process of progression of the follicles from the non-gonadotropin sensitive to the gonadotropin sensitive stages.

Shiomi-Sugaya et al. observed that in an “*in vitro*” model of secondary follicles from mice isolated in gel media, the growth rate of the follicles or their time to atresia correlate with IGF-1 mRNA expression (13). Also interesting is the relationship between IGF-1 mRNA and the presence of theca cells. Follicular progression was arrested by blocking IGF-1 production and restored by the co-culture with the cytokine, thus confirming the importance of IGF-1 in follicular development. These observations suggest a peri-follicular microenvironment where theca cells, beyond providing precursors for local estrogen production, modulate follicular progression through paracrine action of androgens and IGFs.

A completely different approach to study GH role on follicular dynamics is based in modifying GH secretion at the pituitary level (14). In GH df/df Ames dwarf mice GH pituitary secretion is practically abolished. In this context the pool of primordial follicles is clearly increased as compared to N/df or wild type animals. GH administration reverses this situation and decreases primordial follicular count while increasing the number of antral follicles. On the contrary, transgenic mice overexpressing GH have a reduced number of primordial follicles as compared to controls. It is relevant to note that, in the wild type animals, the administration of GH diminishes the primordial follicular population but do not increase the number of antral structures, probably due to a subsequent increased atresia rate. Taken together, these findings suggest that in the absence of GH, follicles remain in the primordial stage.

## Evidence from “Human Models”

In humans the evaluation of GH role on reproduction can be approached through two different models: GHRH or GH receptor mutations or combined pituitary hormone deficiencies (CPHD). In the Itabaianinha County, in Brazil, there is an ethnic group with high prevalence of a mutation of the gene encoding GHRHR gene, resulting in a severe reduction in GH signaling. Beyond the phenotypic characteristics of GH deficiency, the affected individuals have delayed puberty, but are fertile mimicking what is found in animal models with GH deficiency (15).

Similar clinical findings are observed in a group of predominantly Sephardi Jewish with up to 29 mutations of the gene encoding for GHR (16). A cohort of seven married women has been followed for their entire reproductive lives. Five of them have conceived 11 term pregnancies and 4 miscarriages. All pregnancies were spontaneous except for one that was obtained by IVF. Six women reached menopause between 48 and 51 years. In a different model of childhood onset CHPD, Correa reports on five cases from a single center. Pregnancy has been obtained in all cases with controlled ovarian stimulation and GH –LT4 co-treatment (17).

## Summary: Lessons Learned From Animal and Human Models

All experimental or clinical situations of early GH/IGF deprivation result in a delay in pubertal development with a corresponding prolongation of reproductive life. Microscopically there is a change in the composition of the follicular pools with a predominant population of primordial and primary follicles and the absence or limited presence of more advanced stages of follicles. This situation can be reverted with GH or IGF-1 administration. IGF-1 has proven to be necessary for ovulation to occur while in the absence of GH follicular development, ovulation, and pregnancy can take place. In these cases, however, the size of the litter is significantly decreased. All these findings suggest a significant role of both systemic and local GH/IGF-1 regulation in the progress of follicles from non-gonadotropin to gonadotropin dependent status and also in improving follicular development and oocyte maturation. Thus, it is biologically plausible that GH administration can play a positive role in increasing the number of recruited follicles, especially in cases with limited ovarian reserve. If the evidence from animal models can be directly translated into humans, the administration of GH with the purpose of improving the oocyte yield should begin earlier than the stimulation with gonadotropins. In the same line of thinking Gleicher and colleagues explore this hypothesis, www.*clinicaltrial.gov (NCT02179255)*, suggesting to initiate HGH at least 6 weeks prior to start of COS.

**Figure 1:**
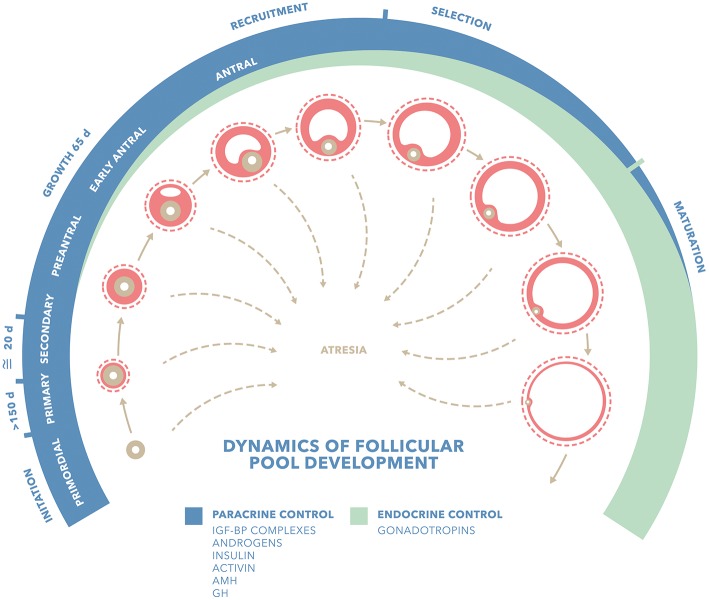
Factors influencing dynamics of follicular pool development. Adapted from Gougeon (18) *IGF-BP complexes (Insulin Growth Factor Binding Protein Complexes); GH (Growth Hormone); AMH (Anti Müllerian Hormone)*. Blue stripe shows follicular stages predominantly influenced by paracrine factors: activators such as GH and IFG-BP complexes, insulin, androgens and activin and AMH. Green stripe shows follicular stages predominantly influenced by endocrine factors such as gonadotropins. Although there has been so far a clear differentiation between gonadotropin-independent and gonadotropin responsive/dependent stages, all these molecules have shown to take part not only at one level, but in the whole folliculo-genesis.

Figure 1 shows factors influencing the dynamics of follicular development. Activators such as GH and IFG-BP (*Insulin Growth Factor Binding Protein)* complexes, Insulin, androgens and activin, might promote follicular growth, transition to antral stage or even follicular recruitment, either by acting as anti-apoptotic factors or enhancing follicular response to gonadotropins. Inhibitors such as *Anti Müllerian Hormone* (AMH) are able to block initial follicle recruitment, transition to antral stage or even the gonadotropin-dependent recruitment. Late follicular stages are predominantly influenced by endocrine factors such as gonadotropins: mainly FSH in the recruitment and selection of the leading follicle and LH at later stages and last oocyte maturation and ovulation. Although there has been a clear differentiation between gonadotropin-independent and gonadotropin responsive/dependent stages, all the molecules mentioned have been shown to take part not only at one level, but in the entire process of folliculogenesis. Basic science studies provide biologically plausible data for GH and IGF-1 as key factors for an optimal follicle development. GH may play an activating role, either directly or indirectly, via for instance IGF-1 in the transition from primordial follicles to late antral stages.

## First Clinical Evidence of Therapeutic GH in IVF

In the late eighties, a few case studies in patients undergoing COS for IVF and ovulation induction (OI) suggested that administration of GH improved the ovarian response to stimulation with gonadotropins (19–21). Later it was shown that GH treatment was associated with minor adverse reactions, mainly gastrointestinal symptoms, in ~17% of cases (8/48) (22). The first reported mechanism by which GH would enhance FSH action was by up-regulating the synthesis of IGF-1 in granulosa cells (23). Animal studies suggested that GH increased the intra-ovarian synthesis of the IGF-1 *in vivo* and *in-vitro* (24, 25) and that this interaction was an important part of ovarian physiology in humans (26, 27). Addition of IGF-1 in granulosa cell cultures increased the intrinsic action of gonadotropins by enhancing aromatase activity, estradiol (E2) and progesterone (P) production and LH receptor formation (27, 28) and was able to stimulate follicular development and oocyte maturation (25).

In 1990, a well-designed controlled clinical trial confirmed the synergistic effects of GH in patients undergoing IVF and stimulated with human menotropin gonadotropin (HMG) (21). However, another RCT with 20 suboptimal responder patients concluded that there was no improvement in the ovarian response by adding GH although there was a trend for more developing follicles (*P* = 0.06) (29). Interestingly, a sub-analysis of this study in patients with polycystic ovaries (PCO) showed a significant increase in the number of follicles developed (*P* = 0.04) and the number of oocytes retrieved (*P* = 0.03). The study did not report pregnancy rates and live birth rates (LBR) (29). Another study focusing on polycystic ovarian syndrome (PCOS) patients as a target for GH treatment found favorable responses in terms of serum and follicular IGF-1 concentrations (30). Despite not reporting conclusive clinical results, these and other early studies reported that GH treatment seemed to promote ovarian steroidogenesis and follicular development (22).

## Meta-Analyses: a Need for Further Research

In 2003, a Cochrane review and meta-analysis concluded that the use of GH in COS for IVF was in need of further research (31). The meta-analysis covered studies with GH co-treatment administered in varying dosages (4, 8, and 12 mg) and with intervention performed alongside stimulation start. There were no significant differences in any outcome measure and at any of the dosages used. Following this meta-analysis, five subsequent meta-analyses assessed the clinical use of GH as adjuvant in IVF. The first analysis reported an increase in the clinical pregnancy rates (CPR) and LBR by the administration of GH during COS with gonadotropins in PORs—an absolute increase in CPR by 16% (95% CI: +4 to +28; fixed effects model) (number-needed-to-treat = 6, 95% CI:4–25). Moreover, GH supplementation was associated with a significantly higher proportion of patients reaching embryo transfer (32). Despite this promising result, the total number of cases included in the meta-analysis was too small to reach robust evidence (only 169 patients in a total of 6 RCTs). It is important to stress that from the 2003 Cochrane review to the meta-analysis by Kolibianakis by (32), only one well designed RCT was published in the literature, comparing the use of GH alone as an adjuvant to COS in PORs (33). The study involved 61 PORs patients, and the study group (*n* = 31) received daily GH co-treatment (4 mg subcutaneously) mg from the first day of GnRHa down regulation (day 21 of the preceding cycle) until the day of the ovulation trigger (OT). The control group (*n* = 30) received the same protocol except for the GH co-administration. A numerically higher CPR was achieved in the GH group (12/31) as compared to the control group (6/30). However, this difference did not reach statistical significance. Prior to that two Chinese studies were conducted in PORs and investigating the use of GH (34, 35). Both studies were only available in full-text in Chinese, hence, we did not include them in this review.

Kyrou et al. (36) performed a meta-analysis of RCT‘s which evaluated interventions aiming at increasing pregnancy rates in PORs. The only adjuvant treatment to standard stimulation that appeared to increase the probability of live birth was the addition of GH (OR 5.22, CI: 95% 1.09–24.99). Later, Duffy et al. (37) made a revised update of the Cochrane meta-analysis, including 10 studies with a total of 440 patients. Results demonstrated a statistically significant difference in LBR favoring co-administration of GH in IVF protocols in POR patients without increasing adverse events (OR 5.39, 95% CI 1.89–15.35). Notably, most of the studies included in these meta-analyses led to a potential bias in the results due to poor description of the method of randomization. Moreover, there were significant differences in timing and dose of GH co-administration as well as high heterogeneity in the definition of POR.

In 2015, Yu et al. (38) performed an updated meta-analysis reporting results in line with previous analyses. The results showed a significant improvement in terms of metaphase II oocytes retrieved, number of 2PN obtained and number of embryos available for transfer by GH supplementation in IVF patients. However, no difference was seen as regards CPR.

## The Search for the Most Optimal Stimulation Protocol

A retrospective matched case–control study including 42 patients explored for the first time the effect of GH as an adjuvant in a micro-dose GnRH agonist flare-up protocol. The study group was treated with 3.33 mg GH daily subcutaneously (SC) for 14 days before starting COS (39). The authors did not find differences in any of the reported outcomes, although the small sample size and the retrospective character of the study necessitated a future RCT to draw firm conclusions. A RCT including a total of 141 patients was subsequently performed in GnRH antagonist co-treated Bologna criteria POR patients (40). In this study, GH administration was initiated on day 6 of hMG stimulation in a daily dose of 2.5 mg SC until the day of HCG trigger. The study group had significantly fewer days of stimulation, more oocytes retrieved and better fertilization rates, albeit the authors did not find significant differences in CPR per cycle and LBR per cycle. These results were similar to those previously published in the first RCT using GH during GnRH antagonist co-treatment (41). In 2015, an open label four arm randomized study including a total of 287 POR patients aligned with the ESHRE Bologna criteria aimed at comparing 4 different stimulation protocols (42). All groups were administered GH on day 6 of hMG stimulation in a daily dose of 2.5 mg SC. Patients were randomly allocated to either a long or short GnRH agonist protocol, mini-flare or GnRH antagonist protocol. The long protocol was superior regarding the number of oocytes retrieved and fertilization rate, although no differences were seen in CPR. More recently, Dakhly et al. explored GH adjuvant treatment in the long agonist protocol in a prospective randomized study with 240 patients (43). The intervention group received adjuvant GH co-treatment 2.5 mg s.c (7.5 IU) from day 21 of the previous cycle along with GnRHa, until the day of HCG trigger. Authors found statistically significant differences in terms of number of oocytes collected in favor of GH [(5.4 ± 1.7) vs. 4.3 ± 2.1], but they failed to show statistical differences in LBR in both fresh (17.5 vs. 14.1%) and cumulative frozen embryo transfer cycles (18.3 vs. 14.7%) (43). However, this study was criticized for mainly two shortcomings (44): (i) A mean of 2.4 and 1.6 embryos were transferred in the study group and the control group, respectively, in the fresh cycle, yielding the results difficult to interpret with today's standard of using single embryo transfer and (ii) the luteal phase support with micronized progesterone pessaries 400 mg twice daily seemed suboptimal for the POR patients and the most optimal approach would be with a combination of HCG injections and micronized progesterone pessaries 400 mg three times daily as described by Yovich previously (45) or other methods of ensuring optimal mid-luteal serum *P* levels (46).

## Most Recent Meta-Analyses

Recently, Li et al. (47) performed a meta-analysis including 11 RCT's with a total of 663 patients. A pooled result, using fixed-effects model showed that the CPR and LBR per transfer were significantly higher in the GH group (RR 1.65, 95% CI 1.23–2.22; *p* < 0.001 and RR 1.73, 95% CI 1.25–2.40; *P* < 0.001, respectively). Moreover, the cycle cancellation rate (RR 0.65, 95% CI 0.45–0.94; *P* = 0.02) was significantly lower in GH co-treated cycles. No significant difference was seen in implantation rate (RR 1.05, 95% CI 0.56–1.99; *P* = 0.87). Although co-treatment with GH significantly increased the number of oocytes retrieved and the number of MII oocytes obtained, there was a high heterogeneity between studies regarding these two outcomes (*I*^2^ = 87 and 89%, respectively) (47). The latest meta-analysis regarding the use of GH in COS was also published in 2017 (48). In that analyses all previous articles were included as well as data from the so-called LIGHT study (49). This was a multicenter, double-blind, placebo-controlled trial performed in 10 centers throughout Australia and New Zealand. Authors did not include Bologna or POSEIDON criteria for POR. A GH dose of 12IU was administered from the first day of stimulation in the intervention group. After 4 years of enrollment, the study was stopped prematurely, reporting only 130 patients randomized. The number of patients reaching an oocyte retrieval per randomized cycle was significantly higher in the GH group (62/65 [95.4%] vs. 51/65 [78.5%], OR 5.67, 95% CI 1.54–20.80), however, no differences were reported in the LBR (9/62, [14.5%] vs. 7/51, [13.7%], risk difference 0.8%, 95% CI −12.1 to 13.7%; OR 1.07, 95% CI 0.37–3.10). Unlike other studies, no statistical differences were reported between groups regarding the mean number of oocytes retrieved (5 vs. 4, rate ratio 1.25, 95% CI 0.95–1.66) and the chance of reaching embryo transfer (53/61 [86.9%] vs. 42/51 [82.4%], OR 1.42, 95% CI 0.50–4.00). No differences in embryo quality were found between groups. Results from this study should be interpreted with caution, as it was underpowered due to the few number of patients included.

## Conclusions

The use of GH to enhance follicular response to gonadotropin stimulation has biological plausibility, as shown in animal and human models. The GH/IGF system plays a pivotal role in the regulation of follicular dynamics. Any experimental manipulation reducing the exposure to either GH or IGF lead to an imbalance between the primordial gonadotropin independent and gonadotropin sensitive follicular pools and a subsequent decrease in the size of the litter. However, to date, although a higher number of oocytes has been consistently reported researchers failed to show benefits in terms of LBR with the use of adjuvant GH. The use of GH is definitely “unfinished business” and future trials with bigger sample size need to be more specific as regards inclusion criteria, treatment protocol and GH dose to draw firm conclusions. Until then, it seems that some clinicians would use GH as adjuvant whereas many would not.

## Author Contributions

The style and concept were developed by CD, JC, TH, and PH. CD and JC produced the figure. All authors contributed to writing the manuscript, contributed with critical review, discussions regarding the final content of this review, and accepted the submission of this manuscript for publication.

### Conflict of Interest Statement

TH received honoraria for lectures from Merck and Ferring. PH received unrestricted research grants from MSD, Merck, and Ferring as well as honoraria for lectures from MSD, Merck, Gedeon-Richter, Theramex, and IBSA. PH is a co-founder in the POSEIDON group. The remaining authors declare that the research was conducted in the absence of any commercial or financial relationships that could be construed as a potential conflict of interest.
